# Case Report: A rare case of familial progressive cholestasis type 10 in an adult with heterozygous MYO5B variant

**DOI:** 10.3389/fgstr.2025.1435168

**Published:** 2025-06-04

**Authors:** Zhang Huimin, Wang Yuan, Xu Chuanyan, Chen Jing

**Affiliations:** Department of Infectious Diseases, Hospital of Chengdu University of Traditional Chinese Medicine (TCM), Chengdu, China

**Keywords:** cholestasis, novel mutation, MYO5B, PFIC, BSEP

## Abstract

Progressive familial intrahepatic cholestasis (PFIC) is a group of rare autosomal recessive cholestatic liver diseases that typically manifest in infancy or childhood. It is characterized by intrahepatic cholestasis, jaundice, pruritus, and malabsorption, with potential progression to cirrhosis, liver failure, and hepatocellular carcinoma. Here, we report a 36-year-old Chinese male patient with delayed-onset PFIC who presented with recurrent jaundice and pruritus. Laboratory investigations excluded viral, autoimmune, or neoplastic causes of liver injury. Liver biopsy demonstrated hepatocyte hydropic degeneration and intracanalicular bile thrombi, while genetic testing revealed compound heterozygous variants in the MYO5B gene: c.3604-1G>C and c.1165G>T (p.V389F). The patient exhibited fluctuating bilirubin levels refractory to initial therapies including corticosteroids, ursodeoxycholic acid, cholestyramine, and artificial liver support. However, bilirubin normalization was achieved following adjunctive traditional Chinese medicine therapy after transfer to our institution. This case highlights that genetic etiologies, particularly MYO5B-related disorders, should be considered in patients presenting with recurrent hyperbilirubinemia, pruritus, and hepatosplenomegaly after excluding common causes (viral, autoimmune, drug-induced, or tumor-related). Genetic testing for MYO5B mutations is warranted in cases of high bilirubin with normal/mildly elevated GGT levels, as early recognition is critical for timely intervention.

## Introduction

Progressive familial intrahepatic cholestasis (PFIC) is a rare group of autosomal recessive disorders caused by mutations in genes encoding proteins predominantly expressed in the canalicular membranes of hepatocytes (1). These disorders are characterized by impaired bile synthesis and transport due to intrahepatic cholestasis (2).

PFIC typically manifests during the neonatal period or infancy, with clinical features including jaundice, pruritus, and hepatosplenomegaly. The disease progresses rapidly, often leading to liver failure. Laboratory findings commonly reveal elevated levels of bilirubin, total bile acids (TBA), alanine aminotransferase (ALT), and aspartate aminotransferase (AST), whereas gamma-glutamyl transferase (GGT) levels are usually within the normal range (3).

Currently, PFIC is classified into 12 subtypes (PFIC1–PFIC12) based on the underlying genetic mutations. The primary causative genes include *ATP8B1* (PFIC1), *ABCB11* (PFIC2), *ABCB4* (PFIC3), *TJP2* (PFIC4), *NR1H4/FXR* (PFIC5), *SLC51A* (PFIC6), *USP53*, *KIFP2*, or *KIFP5* (all classified as PFIC7 subtypes), *KIF12* (PFIC8), *ANCHR* (PFIC9), *MYO5B* (PFIC10), *SEMA7A* (PFIC11), and *VPS33B* (PFIC12) (4). However, some patients with PFIC exhibit unidentified genetic mutations, suggesting the existence of additional genotypes. Despite differences in pathophysiology among PFIC subtypes, all forms result in cholestasis and cholestasis-associated pruritus (5, 6).

Due to the clinical heterogeneity of PFIC, diagnosis is often challenging, and genetic testing is recommended to identify pathogenic variants. Among the subtypes, PFIC10 is primarily caused by mutations in the MYO5B gene, located on chromosome 18q21.1. This gene spans approximately 372 kb and comprises 40 exons, encoding myosin VB (Myo5B), a protein consisting of 1,848 amino acids. The age of onset for PFIC10 varies, with most cases presenting in infancy, though some may manifest in adulthood (7).

We report the case of a 36-year-old male patient who presented with pruritus, jaundice, and splenomegaly as the primary clinical manifestations. Genetic testing identified a rare pathogenic variant in the MYO5B gene, confirming the diagnosis of PFIC-10. Following a combined regimen of traditional Chinese medicine (TCM) and Western medicine, the patient exhibited significant symptomatic improvement.

## Case report

A 36-year-old male was admitted on November 20, 2023, presenting with jaundice and pruritus persisting for over two months. The patient initially developed fever (peak temperature 38.3°C) accompanied by chills approximately two months prior, which resolved following oral medication at a local clinic. However, three days later, he exhibited progressive jaundice, pruritus, dark urine, and diarrhea. Initial laboratory investigations revealed markedly elevated liver enzymes: ALT 725.5 IU/L (reference range: 5–40 IU/L), AST 108 IU/L (8–40 IU/L), total bilirubin (TBL) 223.9 μmol/L (3.4–17.1 μmol/L), direct bilirubin (DBL) 158.6 μmol/L (0–6.8 μmol/L), γ-GGT 173.6 U/L (11–50 U/L), and alkaline phosphatase (ALP) 108 U/L (52–171 U/L). Subsequent testing at a tertiary hospital showed further deterioration: TBL 232.4 μmol/L, DBL 177.0 μmol/L, indirect bilirubin (IDBL) 55.4 μmol/L, ALT 192 IU/L, and GGT 101 IU/L.

Comprehensive diagnostic workup excluded viral hepatitis, autoimmune etiologies, and malignancy. Abdominal contrast-enhanced CT/MRI demonstrated mild hepatosplenomegaly, portal vein trunk thickening, and a small hypodense lesion in the pancreatic tail. Abdominal ultrasonography revealed gallbladder wall thickening with luminal contraction. Hepatic ultrasonography suggested parenchymal heterogeneity consistent with hepatocellular injury. Liver biopsy confirmed mild chronic inflammation (G1S1-2) with hepatocellular cholestasis, hydropic degeneration, focal necrosis, and portal tract lymphocytic infiltration. Fibrosis staging indicated portal expansion with early septal formation. Immunohistochemistry was negative for HBsAg and HBcAg, with CK7 positivity in bile ducts. Special stains (rhodanine, Prussian blue, PAS, D-PAS) were unremarkable.

During the initial hospitalization, the patient received comprehensive hepatoprotective and anti-cholestatic therapy including: ursodeoxycholic acid capsules, ademetionine 1,4-butanedisulfonate injection, glutathione injection, and magnesium isoglycyrrhizinate injection for liver protection and jaundice reduction; human albumin supplementation for hypoalbuminemia; methylprednisolone and dexamethasone for anti-inflammatory management; and cholestyramine powder for pruritus control. Following discharge, the patient developed recurrent hyperbilirubinemia requiring readmission. The subsequent treatment regimen consisted of: ursodeoxycholic acid capsules, ademetionine 1,4-butanedisulfonate injection, alprostadil infusion, magnesium isoglycyrrhizinate injection, glutathione injection, phenobarbital tablets for refractory jaundice, dexamethasone as anti-inflammatory agent, and adjunctive traditional Chinese medicine. This therapeutic approach resulted in significant clinical improvement with reduction of total bilirubin to 77.8 μmol/L at discharge.

**Figure 1:**
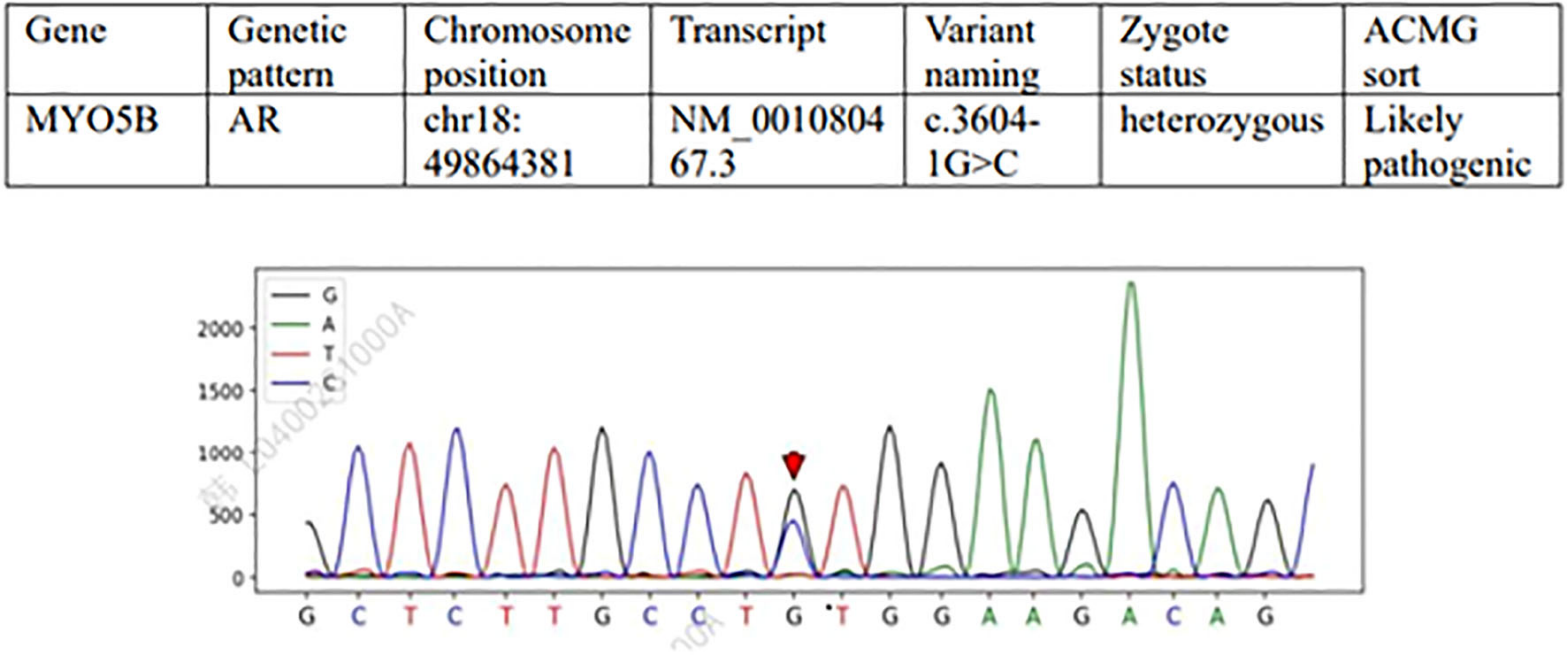
MYO5B gene c.3604-1G>C (Likely pathogenic). Variant type: splice site variation (located in the intronic region, near the exon-intron junction). Sanger sequencing map features: In a normal sequence, the base at this position should be G (reference sequence). The sequencing plot shows that there are doublets of G and C at this location (heterozygous state), indicating that the submitter is heterozygous for G/C at this site. This variant may result in splicing abnormalities (e.g., skipping exons or retaining introns) that affect protein function. Clinical significance: ACMG is classified as “probable” (PVS1+PM2_P), supporting its association with disease (e.g., progressive familial intrahepatic cholestasis type 10).

**Figure 2:**
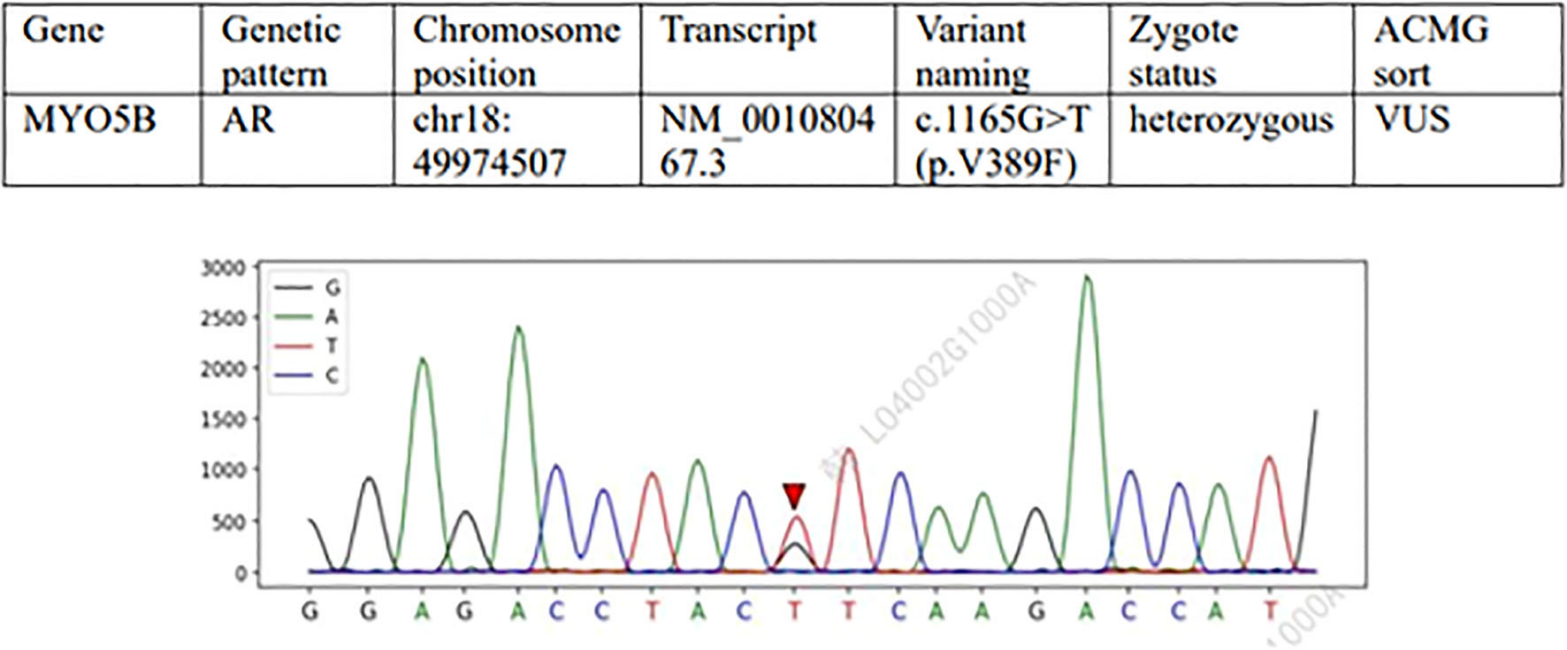
Variation 2: MYO5B gene c.1165G>T (p.V389F) (meaning unknown, VUS). Type of variation: Missense variation (change of nucleotide 1165 from G to T, resulting in change of amino acid 389 from valine [Val] to phenylalanine [Phe]). Sanger sequencing map features: In the normal sequence, this position was G, and the sequencing map showed double peaks of G and T (heterozygosity), confirming that the subject was G/T heterozygosity. This variant may affect protein structure or function (such as hydrophobic changes), but evidence of pathogenicity is insufficient. Clinical significance: ACMG is classified as “of unknown significance” (PM2_P+PP3) and requires further functional testing or family verification (e.g. parental carrying).

Whole genome sequencing analysis during hospitalization revealed a potential compound heterozygous variant at the MYO5B splice site using Targeted Region Capture technology for coding region enrichment and high-throughput sequencing. The detected variants, NM_001080467.3(MYO5B):c.3604-1G>C and NM_001080467.3(MYO5B):c.1165G>T(p.V389F), were annotated according to GRCH38 reference genome and classified following ACMG guidelines with supporting evidence from multiple genomic databases (ClinGen, OMIM, ClinVar, gnomAD, DGV, HGMD). The c.3604-1G>C (**Figure 1**) variant (gnomAD frequency=0) was classified as pathogenic based on PVS1 (predicted loss-of-function splicing variant) and PM2_P criteria, representing a novel unreported mutation. The c.1165G>T missense variant (gnomAD frequency<0.00001) met PM2_P and PP3 (**Figure 2**) criteria (REVEL score=0.651), with in silico predictions suggesting deleterious effects on protein function. These molecular findings showed strong correlation with the patient’s clinical presentation of cholestatic liver disease.

**Figure 3:**
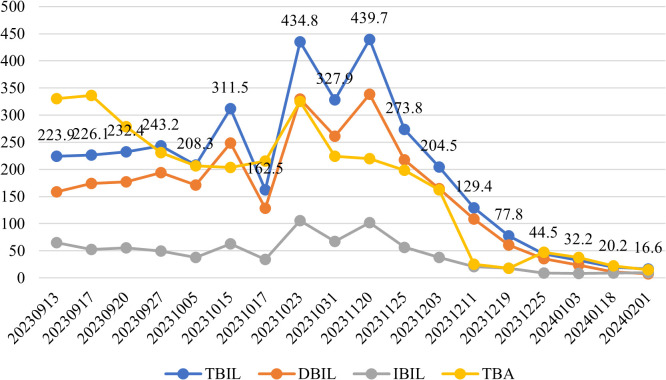
Serial measurements of serum bilirubin and total bile acid concentrations. Blue line:Total bilirubin(TBIL); Orange line:Direct bilirubin(DBIL); Gray line:Indirect bilirubin(IBIL); Yellow line: Total bile acids(TBA).

**Figure 4:**
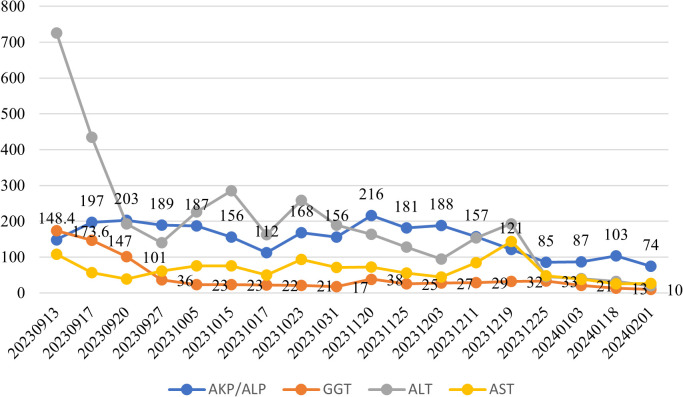
Longitudinal monitoring of hepatic enzymes: ALT, AST, ALP, and GGT. Blue line: Alkaline phosphatase (AKP/ALP); Orange line: Glutamyltranspeptidase (GGT); Gray line: Alanine aminotransferase (ALT); Yellow line: Aspartate aminotransferase (AST).

Following therapeutic intervention, the patient demonstrated significant clinical improvement with normalization of bilirubin levels and complete resolution of jaundice and pruritus. Longitudinal biochemical monitoring demonstrated progressive improvement, with declining levels of both bilirubin and total bile acids as shown in **Figure 3**, along with decreasing values of liver enzymes including ALT, AST, AKP, and GGT as illustrated in **Figure 4**. This favorable outcome suggests that the identified MYO5B variants may represent a previously unrecognized genetic cause of cholestatic liver disease with potential treatment responsiveness.

## Discussion

This study presents a case of a 36-year-old Chinese male with recurrent jaundice and pruritus. After excluding common causes of liver injury including viral infections, autoimmune diseases, alcoholic liver disease, and drug-induced hepatotoxicity, liver biopsy revealed hepatocellular cholestasis. Laboratory investigations showed elevated TBL, ALT, and AST, while γ-GGT and alkaline phosphatase (AKP) levels remained within normal ranges. These findings raised suspicion of an inherited metabolic liver disorder. Whole genome sequencing identified a heterozygous splice-site variant in the MYO5B gene, leading to a diagnosis of progressive familial intrahepatic cholestasis type 10 (PFIC-10). Given the autosomal recessive inheritance pattern, parental genetic testing was recommended.

MYO5B mutations are associated with diverse clinical phenotypes including PFIC and microvillus inclusion disease (MVID), a congenital diarrheal disorder. These mutations disrupt intracellular transport of brush border proteins in intestinal epithelial cells, resulting in diarrheal symptoms (8, 9). Additionally, studies have demonstrated that MYO5B mutations can induce cholestasis by impairing bile acid transporter proteins in hepatic canaliculi (10). MYO5B encodes myosin Vb, a molecular motor protein critical for polarized protein trafficking in epithelial cells through its interaction with RAB11 and RAB8 GTPases (11). In hepatocytes, RAB11A mediates the apical recycling endosome pathway that transports ABC transporters (including BSEP) from the trans-Golgi network to the canalicular membrane, a process essential for proper biliary secretion (12). The bile salt export pump (BSEP) plays a pivotal role in bile acid efflux and bile flow generation, making it central to cholestasis pathogenesis (13). Normally localized to the canalicular membrane, BSEP dysfunction due to deficient expression or mislocalization can lead to impaired bile secretion and subsequent cholestasis (14). MYO5B mutations may disrupt the apical recycling pathway in hepatocytes, causing BSEP mistrafficking and consequent bile acid retention (15).

The relationship between MYO5B mutations and BSEP expression remains incompletely understood. While some studies report reduced BSEP expression and/or mislocalization in MYO5B-associated cholestasis (16), others demonstrate preserved canalicular localization (17), suggesting additional pathogenic mechanisms beyond BSEP dysfunction. Emerging evidence implicates gut microbiota in bile acid homeostasis through the enterohepatic circulation. Microbial dysbiosis may impair intestinal bile acid reabsorption, potentially contributing to MYO5B-related cholestasis (11), indicating a potential gut-liver axis in disease pathogenesis.

Current PFIC management strategies encompass three main approaches: nutritional support, pharmacotherapy, and surgical interventions (18). Nutritional therapy aims to ensure adequate growth and address malabsorption. Pharmacological options include ursodeoxycholic acid (UDCA), which improves biochemical parameters and pruritus in cholestatic disorders (19), and odevixibat, an ileal bile acid transporter inhibitor effective against PFIC-associated pruritus (20). The pharmacological chaperone 4-phenylbutyrate shows promise in restoring BSEP membrane expression (21), while corticosteroids may upregulate hepatobiliary transporters (BSEP, MRP2) and cytochrome P450 enzymes (22), with demonstrated antipruritic effects (23). Surgical options include partial external biliary diversion and liver transplantation for advanced cases.

In our case, early diagnosis and intervention prevented progression to cirrhosis or liver failure. The incorporation of traditional Chinese medicine with conventional therapy resulted in complete symptom resolution and biochemical normalization during follow-up. Continued monitoring remains essential.

This case highlights the importance of considering inherited metabolic disorders in patients presenting with cholestasis, pruritus, jaundice, and hepatosplenomegaly, with or without diarrhea. A comprehensive diagnostic approach combining clinical evaluation, metabolic screening, and genetic testing is crucial for accurate diagnosis. Our findings contribute to the understanding of MYO5B pathogenic variants, facilitating prompt genetic diagnosis, family counseling, and prenatal testing opportunities.

## Data Availability

The datasets supporting this study are openly available in Figshare under the following DOIs: Biochemical monitoring data: https://doi.org/10.6084/m9.figshare.29212511.v1MYO5B mutation maps: https://doi.org/10.6084/m9.figshare.29212514.v1.
